# Triazolopyrimidine herbicides are potent inhibitors of *Aspergillus fumigatus* acetohydroxyacid synthase and potential antifungal drug leads

**DOI:** 10.1038/s41598-021-00349-9

**Published:** 2021-10-26

**Authors:** Y. S. Low, M. D. Garcia, T. Lonhienne, J. A. Fraser, G. Schenk, L. W. Guddat

**Affiliations:** 1 School of Chemistry and Molecular Biosciences, The University of Queensland, Brisbane, QLD 4072 Australia; 2 Australian Infectious Diseases Research Centre, The University of Queensland, Brisbane, QLD 4072 Australia

**Keywords:** Biochemistry, Chemical biology, Drug discovery, Microbiology, Structural biology, Infectious diseases

## Abstract

*Aspergillus fumigatus* is a fungal pathogen whose effects can be debilitating and potentially fatal in immunocompromised patients. Current drug treatment options for this infectious disease are limited to just a few choices (e.g. voriconazole and amphotericin B) and these themselves have limitations due to potentially adverse side effects. Furthermore, the likelihood of the development of resistance to these current drugs is ever present. Thus, new treatment options are needed for this infection. A new potential antifungal drug target is acetohydroxyacid synthase (AHAS; EC 2.2.1.6), the first enzyme in the branched chain amino acid biosynthesis pathway, and a target for many commercial herbicides. In this study, we have expressed, purified and characterised the catalytic subunit of AHAS from *A. fumigatus* and determined the inhibition constants for several known herbicides. The most potent of these, penoxsulam and metosulam, have *K*_*i*_ values of 1.8 ± 0.9 nM and 1.4 ± 0.2 nM, respectively. Molecular modelling shows that these compounds are likely to bind into the herbicide binding pocket in a mode similar to *Candida albicans* AHAS. We have also shown that these two compounds inhibit *A. fumigatus* growth at a concentration of 25 µg/mL. Thus, AHAS inhibitors are promising leads for the development of new anti-aspergillosis therapeutics.

## Introduction

Aspergillosis is an invasive fungal disease caused by members of the genus *Aspergillus. A. fumigatus* makes up 90% of such infections, and in immunocompromised patients these can prove to be debilitating and potentially fatal^1^. Azoles such as voriconazole and itraconazole are the first choice of drugs for treatment, but options are limited due to commonly occurring side effects (e.g. liver toxicity and neuropathy)^2,3^. In addition, the effectiveness of the currently available drugs is being impeded by the emergence of drug-resistant strains^3^. This is especially true in the recent COVID-19 pandemic, where a hybrid *Aspergillus* species, *Aspergillus latus*, was isolated from COVID-19 patients and found to have increased drug resistance compared to its parental strains^4^. These issues highlight the urgent need for alternative or more effective treatments for aspergillosis infections.

A potential new antifungal drug target is acetohydroxyacid synthase (AHAS; EC 2.2.1.6), the first enzyme in the branched chain amino acids (BCAA) biosynthesis pathway. Fungal pathogens *Candida albicans* and *Cryptococcus neoformans*, whose AHAS genes have been knocked out, are auxotrophic for isoleucine and valine, and are no longer virulent in infected mice^5,6^. Inhibition of AHAS therefore shuts down the BCAA biosynthesis pathway and prevents protein synthesis, and ultimately stops the growth of these fungal pathogens in mice^5,6^. It is also noteworthy that AHAS and the BCAA pathway are absent in animals and humans. Therefore, inhibitors that are designed to target AHAS are likely to be harmless to these species.

**Figure 1:**
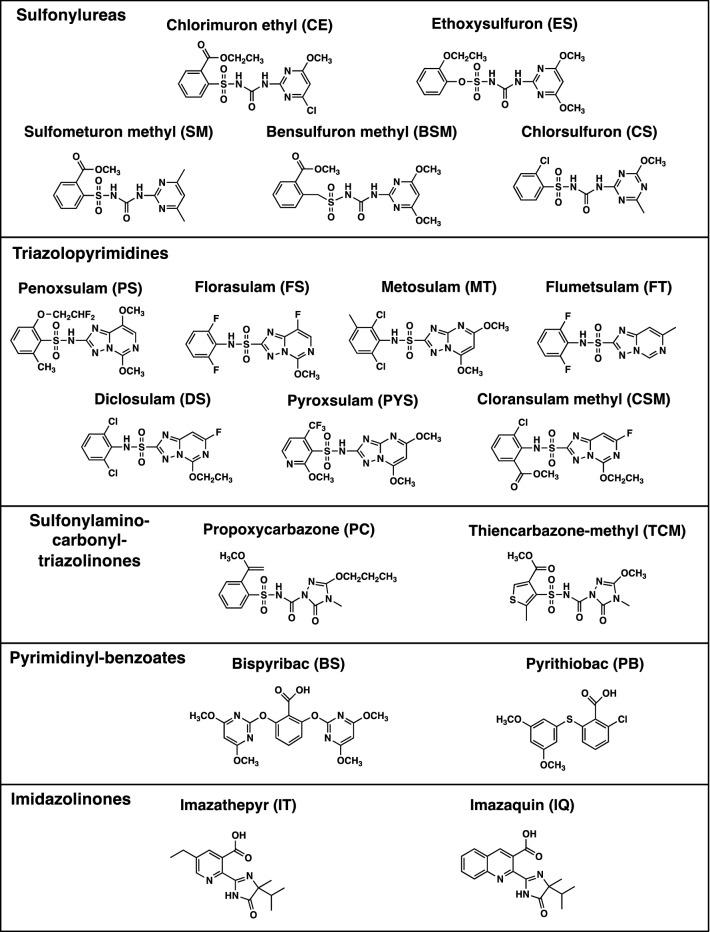
Chemical structures of the commercial herbicides evaluated here. A general feature of the sulfonylureas, triazolopyrimidines, and sulfonylamino-carbonyl-triazolinones is the presence of both an aromatic and a heterocyclic ring. The structural images were created using CHEMDRAW 20.0.

AHAS inhibitors have previously been developed into commercial herbicides^7,8^. There are now over 50 such commercial herbicides across five different chemical families, namely the sulfonylureas, triazolopyrimidines, sulfonylamino-carbonyl-triazolinones, pyrimidyl(oxy/thio)benzoates and imidazolinones (Fig. 1). These herbicides have been used with great efficacy for crop protection worldwide, and importantly exhibit low toxicity toward animals^9^. AHAS inhibition by herbicides follows an elaborate process where two mechanisms are involved. Beside direct inhibition of enzyme activity, most herbicides are able to trigger the oxidative inactivation of the enzyme in a process referred to as “time-dependent accumulative inhibition”^10,11^. The latter process is an important contributor to the potent herbicidal^10,12^ and anti-fungal^13,14^ activity exhibited by these compounds.

Here it is suggested that these herbicides could also possess anti-aspergillosis activity. In support of this hypothesis, several AHAS inhibitors have been shown to inhibit the growth of human fungal pathogens in culture and in mice^13,14^. Indeed, members of the sulfonylurea family of herbicides can inhibit *C. albicans* AHAS (*Ca*AHAS) and *C. albicans* growth^14^. In addition, a novel series of triazolopyrimidines also possesses antifungal properties against *A. fumigatus* grown in culture (although these compounds were not directly tested as AHAS inhibitors)^15^. In this study, we expressed, purified and characterised the catalytic subunit of recombinant *A. fumigatus* AHAS (*Afu*AHAS) and evaluated the inhibitory properties of commercial herbicides from all five chemical families. We also assessed the effectiveness of these compounds on *Aspergillus* cell growth in culture, demonstrating their potential as drug leads to combat aspergillosis.

## Results and discussion

### Protein expression and purification

**Figure 2:**
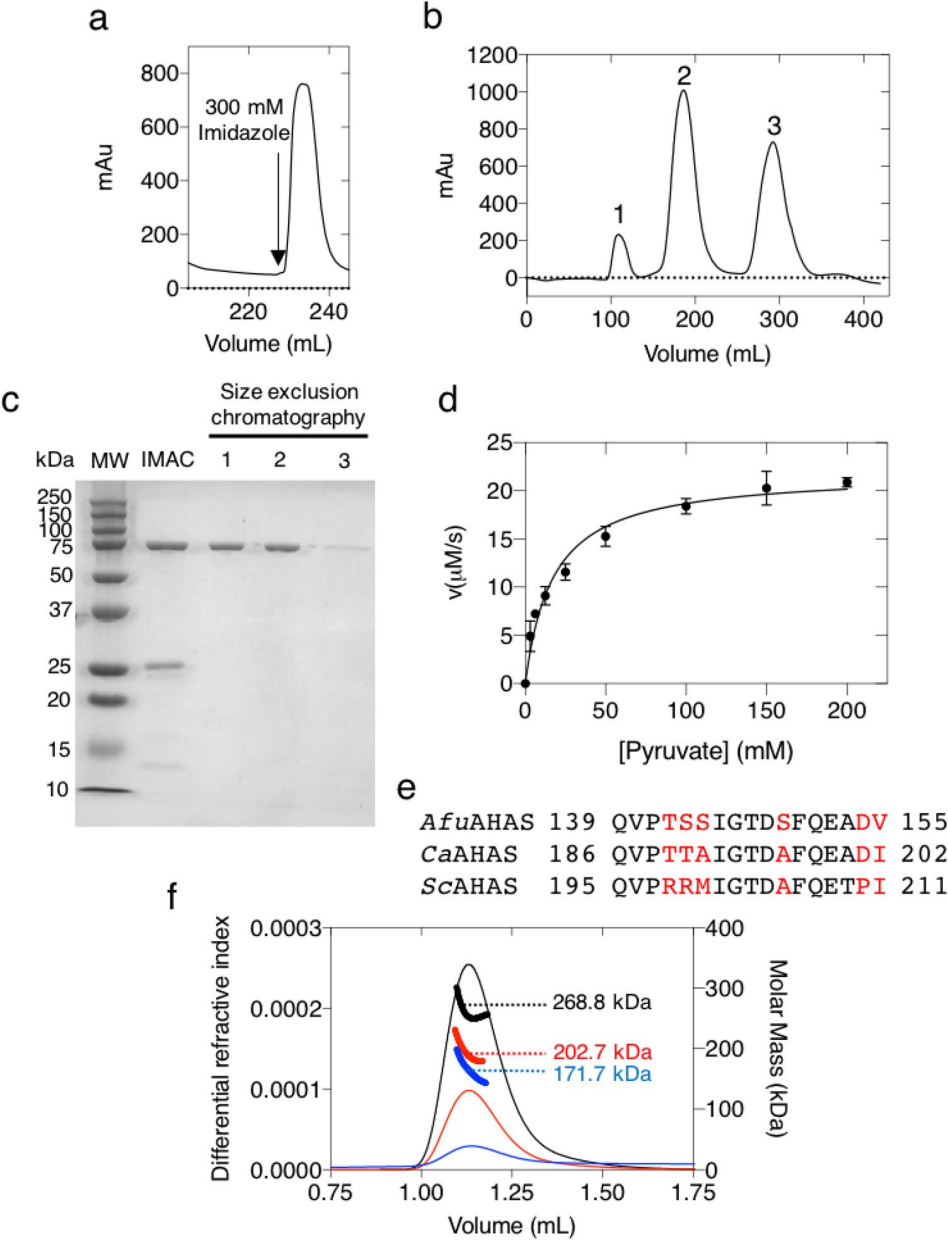
Purification of *Afu*AHAS and determination of its quaternary structure. (**a**) IMAC purification from cell lysate. (**b**) Size exclusion chromatography of the IMAC eluate using a Sephacryl-200-HR column (Pharmacia). Peak 1 corresponds to the void volume. Peak 2 has enzymatic activity. Peak 3 represents the cofactor salts. (**c**) Cropped SDS-PAGE for the purification of *Afu*AHAS. *MW* Molecular weight marker, IMAC eluate; Lanes 1, 2 and 3 correspond to peaks 1, 2 and 3 of the size exclusion chromatogram. See Supplementary Fig. 1 for uncropped version. (**d**) Pyruvate saturation curve for *Afu*AHAS. The data (measured in triplicate) were obtained using a continuous spectrophotometric method. The solid curve shows the best fit to the Michaelis–Menten equation with an R^2^ value of 0.97. (**e**) Comparison of the Q-loop sequences in the catalytic subunit between *Afu*AHAS, *Ca*AHAS and *Sc*AHAS. (**f**) SEC-MALS of peak 2 at three different loading concentrations (1, 5, 10 mg/mL, in blue, red and black, respectively) using a Superdex 200 Increase 5/150 GL column. The MALS data were processed using the ASTRA software (Wyatt Technology).

*Afu*AHAS was recombinantly expressed (see “Materials and methods”), and purified by IMAC (Fig. 2a) and size exclusion chromatographies (Fig. 2b). SDS-PAGE showed a protein of ~ 75 kDa (Fig. 2c), in good agreement with the calculated molecular mass (72 kDa) of the *Afu*AHAS catalytic subunit.

### Characterisation of *Afu*AHAS

The catalytic activity of *Afu*AHAS using pyruvate as a substrate was determined by the continuous method. The data were fitted using the Michaelis–Menten equation (R^2^ = 0.97, Fig. 2d). The *K*_*M*_ for pyruvate for *Afu*AHAS is 17.32 ± 1.97 mM, and the *k*_*cat*_ is 3.29 ± 0.10 s^−1^. When comparing these kinetic parameters to that of *Ca*AHAS (*K*_*M*_*:* 2.6 ± 0.1 mM, *k*_*cat*_: 3.75 ± 0.13 s^−1^)^14^ and *Sc*AHAS (*K*_*M*_*:* 3.6 ± 0.6 mM, *k*_*cat*_: 2.4 ± 0.25 s^−1^)^14,16^, the *K*_*M*_ of *Afu*AHAS is ~ four to six fold higher, suggesting the active site structure and response to substrate binding are not identical. The varying *K*_*M*_ values may be attributed to the sequence differences in the catalytic Q-loop, whose presence is necessary for catalysis to occur (Fig. 2e). Further insights on the structure and dynamics of the active sites during catalysis will be required to understand the effects of these differences.

In order to determine the number of subunits in *Afu*AHAS, size exclusion chromatography (SEC) followed by multi-angle light scattering (MALS) was performed (Fig. 2f). From the MALS analysis, the 1, 5, and 10 mg/mL samples have molecular masses of 171.7, 202.7 and 268.8 kDa, respectively. The value of 268.8 kDa is consistent with the theoretical molecular mass for a tetramer of 288.3 kDa. These results suggest that at higher enzyme concentrations, *Afu*AHAS is predominantly tetrameric, but upon dilution the presence of lower molecular weight forms (*e.g.* dimeric proteins) becomes more relevant. Previous studies have shown that the catalytic subunits of *Saccharomyces cerevisiae* AHAS crystallize as dimer^17^, while *A. thaliana* and *C. albicans* AHAS catalytic subunits crystallize as tetramers^13,18^. However, in general AHAS complexes adopt the shape of a Maltese cross where the active form of the CSU is dimeric^19^. This suggests that the tetrameric form of the catalytic subunit observed at higher concentrations in *A. thaliana* and *C. albicans* crystal structures^12,13,18^, and here through SEC-MALS of *Afu*AHAS, may not represent a physiologically active form of the enzyme.

### *K*_*i*_ values of commercial herbicides for *Afu*AHAS

**Figure 3:**
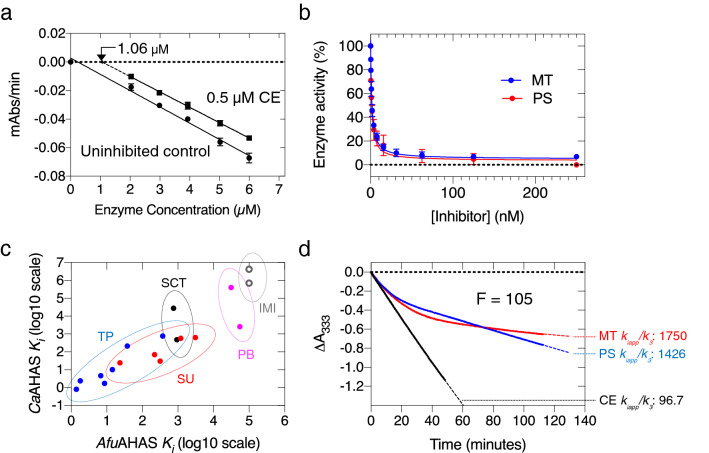
Inhibition of *Afu*AHAS by commercial herbicides. (**a**) Measurement of *Afu*AHAS activity in the presence and absence of 0.5 µM CE to determine herbicide binding stoichiometry. Extrapolation of the linear regression line (dashed line) showed that 1.06 µM of enzyme was fully inhibited by 0.5 µM CE. (**b**) *Afu*AHAS catalytic activity *vs.* inhibitor concentration. The data were fitted using Eq. (1) (see “Materials and methods”) for tight binding inhibitors. (**c**) Correlation of *K*_*i*_ values between *Afu*AHAS and *Ca*AHAS for the five AHAS herbicide families. TP: Triazolopyrimidines, SU: Sulfonylureas, SCT: Sulfonylamino-carbonyl-triazolinones, PB: Pyrimidinyl-benzoates, IMI: Imidazolinones. **d.** Accumulative inhibition curves for PS, MT and CE. The data were fitted using Eq. (3) (dashed line). For a direct comparison of the strength of accumulative inhibition, the F value (free enzyme/enzyme-inhibitor complex ratio) was set to 105 for the three inhibitors. The data show that MT is the most potent accumulative inhibitor. Images were created using GraphPad Prism 7.01.

The herbicide binding stoichiometry of *Afu*AHAS was investigated by measuring the activity of increasing concentrations *Afu*AHAS in the absence and presence of 0.5 µM of the sulfonylurea, chlorimuron ethyl (CE; Fig. 1). The results show that 0.5 µM CE can fully inhibit 1.06 µM of *Afu*AHAS (Fig. 3a), giving a 1:2 inhibitor to enzyme ratio, which has also been observed in other AHAS studies^10^. Thus, once one active site is blocked, communication is halted and the second active site is no longer capable of substrate turnover^20^.

Eighteen commercial herbicides from five chemical families (Fig. 1) were tested for their ability to inhibit *Afu*AHAS. In order to accurately determine the *K*_*i*_ values of herbicides for *Afu*AHAS, a partial anaerobic environment was established through nitrogen bubbling of the assay buffer in the presence of 2-mercaptoethanol (see “Materials and methods”, Supplementary Fig. 2)^12^.

The *K*_*i*_ values of the herbicides for *Afu*AHAS under these conditions are listed in Table 1. Among the five chemical families, the triazolopyrimidines generally have the highest affinity. PS and MT have the lowest *K*_*i*_ values of 1.8 ± 0.92 nM and 1.4 ± 0.18 nM, respectively (Fig. 3b). For the triazolopyrimidine family there is a strong correlation in *K*_*i*_ values for *Afu*AHAS and *Ca*AHAS (Table 1), suggesting the herbicides of this family have very similar binding modes in the two enzymes (discussed below).

**Table 1:** Apparent first-order rate of inhibition (*k*_*iapp*_), first-order rate of enzyme recovery (*k*_*3*_), and inhibition constants (*K*_*i*_*)* of the commercial herbicides for *Afu*AHAS.

Herbicide	*K*_*i*_ (nM)	*K*_*i*_ for *Ca*AHAS (nM)*	*k*_*iapp*_ (min^−1^)	*k*_*3*_ (min^−1^)	*k*_*iapp*_*/k*_*3*_
**Sulfonylureas**					
CE	23.4 ± 1.3	24.3 ± 2.5	0.6 ± 0.1	0.006	96.7
ES	220 ± 11	69.3 ± 6.9	1.0 ± 0.3	0.031	31.3
SM	3100 ± 0.5	630 ± 51	0.2 ± 0.1	0.0065	26.2
CS	1200 ± 0.2	570 ± 59	NAI	NAI	−
BSM	316 ± 101	29.9 ± 2.7	0.7 ± 0.1	0.0026	257.7
**Imidazolinones**					
IQ	> 10^5^	716,000 ± 61,000	ND	ND	−
IT	> 10^5^	4.3 ± 0.7 × 10^6^	ND	ND	−
**Triazolopyrimidines**					
PS	1.8 ± 0.9	2.4 ± 0.3	18.5 ± 2.6	0.013	1426
FS	14.4 ± 2.7	10.1 ± 1.7	17.8 ± 0.6	0.014	1268
MT	1.4 ± 0.2	0.8 ± 0.1	4.2 ± 0.6	0.0024	1750
FT	373 ± 70	773 ± 62	NAI	NAI	−
DS	8.7 ± 5.0	1.7 ± 0.8	3.2 ± 0.8	0.006	530
PYS	37.8 ± 1.5	212 ± 18	6.9 ± 0.4	0.05	138.8
CSM	6.8 ± 3.4	4.6 ± 1.2	8.7 ± 1.8	0.016	545
**Sulfonylamino-carbonyl-triazolinones**					
PC	937 ± 460	484 ± 37	0.34 ± 0.09	0.03	11.3
TCM	752 ± 86	28,000 ± 6000	0.59 ± 0.03	0.011	53.6
**Pyrimidinyl-benzoates**					
BS	55,000 ± 1400	2600 ± 300	NAI	NAI	–
PB	31,100 ± 2000	403,000 ± 37,000	NAI	NAI	–

*K_*i*_ values for *Ca*AHAS are shown for comparison were obtained from Garcia et al.^13^.

*NAI* No reversible accumulative inhibition observed, *ND* Not determined.

See Fig. 1 for chemical structures of compounds.

Amongst the sulfonylureas, CE has the lowest *K*_*i*_ (23.4 ± 1.3 nM), while the other sulfonylureas studied here have K_i_ values in the range from 220 to 3100 nM (Table 1). Relative to *Ca*AHAS, the sulfonylureas bind to *Afu*AHAS with an increase in *K*_*i*_ of ~ 4–10-fold. For the sulfonylamino-carbonyl-triazolinones and pyrimidinyl-benzoates, the herbicides have *K*_*i*_ values that are generally higher than those of the triazolopyrimidine and sulfonylurea families. Furthermore, the imidazolinones tested here show no inhibition of *Afu*AHAS at concentrations up to 100 µM, whilst they weakly inhibit *Ca*AHAS. Overall, however, there is a good correlation of the *K*_*i*_ values between families and between the two enzymes (Fig. 3c).

### Accumulative inhibition by the commercial herbicides on *Afu*AHAS

Accumulative inhibition of *Afu*AHAS was evaluated through the determination of the apparent first-order rate constants of enzyme inactivation (*k*_*iapp*_) and enzyme recovery (*k*_*3*_)^11^, and the efficiency of accumulative inhibition, *k*_*iapp*_/*k*_*3*_^13^ (Table 1). Among all the herbicides, the members of the triazolopyrimidine family were generally shown to be most efficient accumulative inhibitors of *Afu*AHAS (Table 1). In particular, PS and MT have the most potent time-dependent inhibition (*k*_*iapp*_/*k*_*3*_ values of 1426 and 1750, respectively), reflected by the high *k*_*iapp*_ of accumulative inhibition and the relatively low rate of enzyme recovery (*k*_*3*_) (Table 1). PS has a ~ four-fold higher rate of enzyme inactivation compared to MT. However, due to its ~ five-fold lower enzyme recovery rate, stronger accumulative inhibition is achieved by MT over time (Fig. 3d). Comparison of the *k*_*iapp*_ and *k*_*3*_ values for PS and MT in *Afu*AHAS and *Ca*AHAS show that the *k*_*iapp*_ values are similar between the two enzymes (*Afu*AHAS *k*_*iapp*_: 18.5 min^−1^ (PS), 4.2 min^−1^ (MT); *Ca*AHAS *k*_*iapp*_: 15.26 min^−1^ (PS), 3.39 min^−1^ (MT)^13^), while the *k*_*3*_ values are ~ five to ten-fold lower in *Afu*AHAS for PS and MT, respectively (*Afu*AHAS *k*_*3*_: 0.013 min^−1^ (PS), 0.0024 min^−1^ (MT); CaAHAS *k*_*3*_: 0.074 min^−1^ (PS), 0.023 min^−1^ (MT)^13^). Consequently, the accumulative inhibitory efficiencies of PS and MT for *Ca*AHAS (*k*_*iapp*_/*k*_*3*_: 206 and 147.4 for, respectively^13^) are approximately one order of magnitude smaller than for *Afu*AHAS. Thus, taking into account both the corresponding *K*_*i*_ values and accumulative inhibition, PS and MT are more potent inhibitors of *Afu*AHAS than *Ca*AHAS.

### Analysis of herbicide binding in *Afu*AHAS

**Figure 4:**
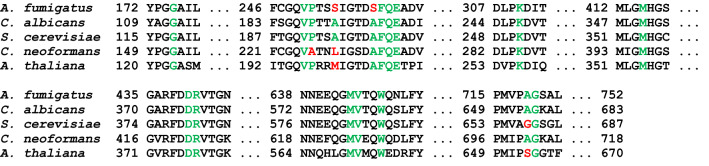
Partial alignment of four fungal and one plant AHAS sequences highlighting the herbicide binding site residues. The conserved residues are in green, while the variants are in red.

**Figure 5:**
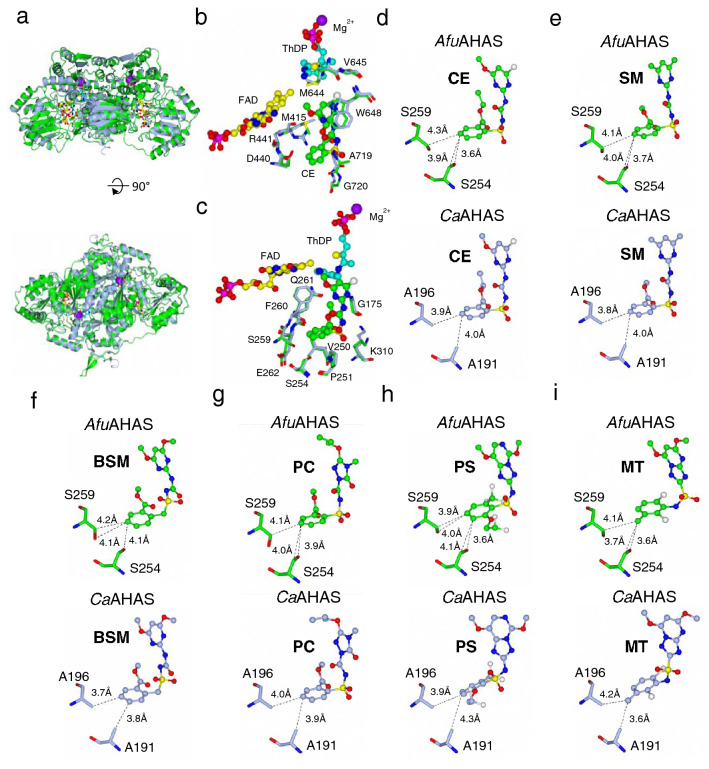
Structural comparisons of *Afu*AHAS and *Ca*AHAS. (**a**) The overall structure of the *Afu*AHAS homology model (green) superimposed onto the crystal structure of *Ca*AHAS (light blue). (**b**, **c**) The herbicide binding sites in *Afu*AHAS and *Ca*AHAS. Residues that make up the binding site from the two different subunits are shown separately in panels (**b**) and (**c**). (**d–i**) Comparison of CE, SM, BSM, PC, PS and MT binding to S254 and S254 in the *Afu*AHAS model and A191 and A196 of *Ca*AHAS in the crystal structures (PDB IDs for the structures of the complexes are 6DEL, 6DEP, 6DEM, 6DES, 6DEQ and 6DER, respectively). The herbicides, thiamine diphosphate and flavin adenine dinucleotide are shown as ball and stick models with the carbon atoms in blue or green for the herbicides and protein and cyan and yellow for thiamine diphosphate and flavin adenine dinucleotide. The magnesium ions are shown as purple spheres. The amino acid side-chains are shown as stick models. The dashed lines represent distances between the two atoms, not necessarily bond formation. The images were prepared using CCP4mg^22^.

Analysis of *Afu*AHAS, *S. cerevisiae* AHAS (*Sc*AHAS), *Ca*AHAS, *C. neoformans* AHAS (*Cn*AHAS) and *At*AHAS catalytic subunit sequences shows that most of the herbicide binding site residues are highly conserved amongst these AHASs (Fig. 4). The exceptions are S254 and S259 in *Afu*AHAS, which are hydrophobic residues in the other fungal and plant AHASs, as well as A719, which is a glycine or serine in *Sc*AHAS and *At*AHAS, respectively (Fig. 4). The conservation of herbicide binding site residues in *Afu*AHAS and *Ca*AHAS is consistent with the correlation found in the *K*_*i*_ values (Fig. 3c). Attempts to crystallize *Afu*AHAS, either in the presence or absence of inhibitors were not successful, so molecular modelling and docking was used to provide structural explanations for the inhibition results. A homology model of *Afu*AHAS was generated using SWISS-MODEL^21^ and the crystal structures of *Ca*AHAS with bound herbicides were used to identify herbicide interactions in *Afu*AHAS (Fig. 5). The homology model has an rmsd of 0.368 Å for 1196 out of 1231 Cα atoms after superimposition with *Ca*AHAS. In total, 95.7% of the amino acid residues have favourable Ramachandran dihedral angles and there are 0.4% outliers. The modelling shows that the herbicide binding site structures in *Ca*AHAS and the *Afu*AHAS model are similar (Fig. 5a–c). The most significant differences are A191 and A196 in *Ca*AHAS which are replaced by serine (*i.e.* S254 and S259; Figs. 4 and 5).

Based on the published *Ca*AHAS structures, A191 and A196 form hydrophobic interactions with the aromatic ring (Fig. 1) of the herbicides (Fig. 5)^13^. Inhibitor docking with the *Afu*AHAS model shows little change in the distances of S254 and S259 to the bound herbicide compared to A191 and A196 in *Ca*AHAS (Fig. 5). The proximity of the serine residues to the bound herbicides remain sufficient for hydrophobic contacts to occur. The change in *K*_*i*_ values (Table 1) observed when sulfonylureas and sulfonylamino-carbonyl-triazolinones bind could be the result of the subtle changes to the herbicide binding site in *Afu*AHAS, *e.g.* the serine residues might promote binding of an ordered water molecule, thereby modifying the binding mode of the inhibitors. However, for PS and MT these sequence changes have no apparent effect on binding.

The presence of S254 and S259, not observed in the other pathogenic fungi and *A. thaliana*, could provide a target for the design of specific *A. fumigatus* inhibitors. Introducing hydrophilicity into the aromatic ring structure of the herbicide to form hydrogen bonds with the serine residues may potentially improve inhibitor binding affinity.

### Minimum inhibitory concentration of *Aspergillus* sp

Lee et al. have shown that sulfonylurea herbicides have antifungal activity that reduces the growth of *C. albicans* in culture, while Garcia et al*.* have shown that CE can reduce *C. albicans* growth in infected mice^13,14^. In this study, the minimal inhibitory concentration (MIC) of the herbicides on the cell growth was determined for four pathogenic *Aspergillus* species^23^, *A. fumigatus*, *A. nidulans*, *A. niger*, *A. flavus*. The results show that CE, PS and MT inhibit the growth of *A. fumigatus* at 100, 25 and 25 µg/mL, respectively (Table 2). PS and MT also inhibit *A. nidulans* growth at 25 µg/mL. None of the other compounds tested showed activity at < 100 µg/mL, and the growth of *A. niger* and *A. flavus* is not impaired by any of the herbicides listed in Table 2.

**Table 2:** MIC values of herbicides for *A. fumigatus*, *A. nidulans*, *A. niger* and *A. flavus* cells.

Herbicide	*A. fumigatus*	*A. nidulans*	*A. niger*	*A. flavus*
	MIC (µg/mL)^a^			
**Sulfonylureas**				
CE	100	> 100	> 100	> 100
ES	> 100	> 100	> 100	> 100
SM	> 100	> 100	> 100	> 100
CS	> 100	> 100	> 100	> 100
BSM	> 100	> 100	> 100	> 100
**Triazolopyrimidines**				
PS	25	25	> 100	> 100
FS	> 100	> 100	> 100	> 100
MT	25	25	> 100	> 100
FT	> 100	> 100	> 100	> 100
DS	> 100	> 100	> 100	> 100
PYS	> 100	> 100	> 100	> 100
CSM	> 100	> 100	> 100	> 100
**Sulfonylamino-carbonyl triazolinones**				
PC	> 100	> 100	> 100	> 100
TCM	> 100	> 100	> 100	> 100
**Pyrimidinyl-benzoates**				
BS	> 100	> 100	> 100	> 100
PB	> 100	> 100	> 100	> 100
**Imidazolinones**				
IT	> 100	> 100	> 100	> 100
IQ	> 100	> 100	> 100	> 100

^a^MIC indicates concentration of herbicides in wells with no observable *Aspergillus* growth.

**Figure 6:**
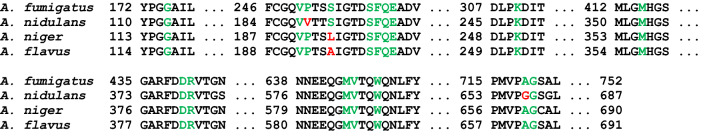
Partial alignment of *A. fumigatus*, *A. nidulans*, *A. niger* and *A. flavus* AHAS sequences highlighting the herbicide binding site residues. Herbicide binding site residues are identified by red or green text to indicate variable or identical sequences, respectively.

The difference in susceptibility of the four *Aspergillus* species to the same herbicide (Table 2) may be attributed to a number of factors. However, comparison of the herbicide binding site residues between the different *Aspergillus* species (Fig. 6) show few differences, suggesting binding to the enzyme is not one of these. The lack of inhibitory activity on *A. niger* and *A. flavus* growth may be due to the production of proteins that manage oxidative stress, or differentially metabolize these herbicides (e.g. P_450_s)^24^.

Richie et al*.* showed that *A. fumigatus* can scavenge BCAA from sheep blood, raising concerns on the efficacy of inhibiting the BCAA biosynthesis pathway for this fungus^15^. However, the primary route of *A. fumigatus* infection is through the lungs, where a porcine model has provided evidence that BCAA availability is limited in pulmonary secretions^25^. *A. fumigatus* is thus unlikely to be able to scavenge sufficient BCAAs from the pulmonary environment and would have to rely on de novo synthesis for growth. A biological assay that would closely mimic these conditions would provide a better estimate of the MIC values. In terms of potential toxicity, compounds of the triazolopyrimidine family have been shown to only be cytotoxic at concentrations at > 128 µg/mL on human blood and liver cell lines^15^. The MIC values of PS and MT are at least eight-fold lower than this concentration, suggesting that these herbicides have minimal cytotoxicity for human cells, and thus they are suitable drug leads.

## Conclusion

Evaluation of the five chemical families of commercial herbicides show that the trizaolopyrimidines have potent inhibitory properties against *Afu*AHAS. In particular, the herbicides PS and MT are tight binding inhibitors and also have strong accumulative inhibitory properties. These two herbicides are therefore good starting points for the design of novel antifungal compounds that target *Afu*AHAS. However, the bioavailability of the herbicide in *Aspergillus* cells needs to be improved for the design of more effective compounds that can prevent growth of this fungal pathogen.

## Materials and methods

### Preparation of *Afu*AHAS gene construct

The amino acid sequence for the catalytic subunit of *Afu*AHAS was obtained from the National Centre for Biotechnology Information (NCBI) (NCBI reference sequence: XP_754588.1). The construct was modified to remove the DNA coding for the first 111 amino acids belonging to the mitochondrial transit peptide sequence. An AUG start codon was added at the N-terminus of the peptide sequence. A Tobacco Etch Virus protease restriction site and a hexahistidine tag were added at the C-terminus of the peptide sequence. The gene sequence was synthesised in a pUC57 plasmid by Biomatik (Cambridge, Canada). The coding sequence for *Afu*AHAS was excised from the plasmid by *Hin*dIII/*Nde*I (New England Biolabs) digestion. The gene was then ligated with the pET30A(+) vector cut using the same restriction sites. *Escherichia coli* BL21 (DE3) cells were then transformed using the resultant pET30A(+)—*Afu*AHAS plasmid.

### Protein expression and purification

All reagents were obtained from Sigma-Aldrich (St Louis, MO, USA) and were of analytical grade, unless otherwise stated. Protein expression and purification were performed as described previously for *Ca*AHAS, but with some differences^13^. The gel filtration buffer used for size exclusion chromatography contains 10 mM potassium phosphate buffer pH 7.2, 300 mM NaCl, 10 µM FAD and 1 mM DTT. The fractions containing the folded enzyme were pooled, and the potassium phosphate concentration was increased to 200 mM and 10 mM of MgCl_2_ was added before concentration and storage at − 80 °C.

### *Afu*AHAS assays

*Standard assays.* AHAS activity was measured in standard assay buffer containing 200 mM potassium phosphate buffer at pH 7.2, 10 mM MgCl_2_, 1 mM ThDP, 10 µM FAD and 100 mM pyruvate, at 30 °C.

Two methods were used:

1. The continuous spectrophotometric method^26^. The variation of absorbance at 333 nm wavelength was recorded (using a Shimadzu UV2550 UV/VIS spectrophotometer) to measure the disappearance of pyruvate (ɛ = 17.5 M^−1^ cm^−1^).
2. The colorimetric single point method^27^. Kinetic assays (in 100 µL total volume) were stopped by adding 11 µL of 10% H_2_SO_4_ to give a final concentration of 1% H_2_SO_4_. The product of the enzymatic reaction, 2-acetolactate, was converted to acetoin by incubating the resultant mixture at 60 °C for 15 min. The amount of acetoin was determined by incubation with 120 µL of 0.5% creatine (w/v) and 120 µL of 5% α-napththol (w/v) in 4 M NaOH for 15 min at 60 °C, followed by 10 min incubation on ice for colour development. The samples were then centrifuged at 9335 × g rpm for two minutes to remove any precipitate. 200 µL of the supernatant was aliquoted into a flat bottomed 96-well plate (Pathlength at 200 µL = 0.555 cm) and the A_525_ (absorbance of acetolactate at 525 nm) of the supernatant was measured (ɛ_M_ = 22,700 M^−1^ cm^−1^).

#### Determination of the kinetic parameters (*K*_*cat*_ and *K*_*M*_) of *Afu*AHAS reaction

The continuous spectrophotometric method (see above) was used to determine the *K*_*M*_ of the substrate, pyruvate. The FAD of *Afu*AHAS was fully reduced to remove the lag phase prior to measuring the rates^19^. 100 µL of assay mixture was incubated at 30 °C at varying pyruvate concentrations (0.03–200 mM) containing 6.6 µM *Afu*AHAS. The data were fitted to the Michaelis–Menten equation to obtain the *K*_*M*_ and the *k*_*cat*_.

#### Preparation of partial anoxic buffer

Partial anoxic assay buffer was made by bubbling nitrogen gas through the standard assay buffer for 30 min to purge dissolved oxygen and by including 14.2 µM 2-mercaptoethanol^12^. The combined effect of 2-mercaptoethanol and nitrogen bubbling on accumulative inhibition generated by PS is shown in Supplementary Fig. 2.

#### *K*_*i*_ determination by the colorimetric single point method

49.37 nM *Afu*AHAS was first incubated at 30 °C in partial anoxic assay buffer for 20 min. Next, 90 µL of the assay mixture containing the enzyme was added to 8 µL of nitrogen-gas-treated water containing 2 uL of varying concentrations of inhibitors dissolved in dimethyl sulfoxide (DMSO), including DMSO alone for the control. The mixture was then incubated at 30 °C for 15 min, before stopping the reaction with 10% H_2_SO_4_ and quantifying the amount of 2-acetolactate (see colorimetric method above). The experiments were repeated three times. Equation (1)^28^ was used to fit the data for tight binding inhibitors (CE and all triazolopyrimidines apart from FT) for which the enzyme concentration has to be taken into account. Equation (2)^29^ was used to fit the data for all other herbicides (medium binding inhibitors). V_0_ and V_1_ are the uninhibited rate and inhibited rate respectively, [I] is the total inhibitor concentration and [E] is the total enzyme concentration.

1 $${\text{V}}_{1} = {\text{V}}_{0} * \frac{{ - \left( {[{\text{I}}] - [{\text{E}}] + {\text{Ki}}} \right) + \left( {\left( {[{\text{I}}] - [{\text{E}}] + {\text{Ki}}} \right)^{2} + 4 * [{\text{E}}] * {\text{Ki}}} \right)^{0.5} }}{{2 * [{\text{E}}]}}$$

2 $${V}_{1} = \frac{{V}_{0}}{\text{1} + \frac{\left[{\text{I}}\right]}{\text{Ki}}}+ \text{c}$$

#### Accumulative inhibition assay

The continuous spectrophotometric method (see above) was used where 2.6 µM *Afu*AHAS was incubated with the standard assay buffer at 30 °C for 20 min to reach maximum enzyme activity before addition of inhibitor. 25 nM of PS, FS, and MT, and 100 nM of the other herbicides were used in the assay. The accumulative inhibition induced by these herbicides was observed for 45 min. All experiments were repeated three times. The data were fitted to Eq. (3)^10,11^ to calculate the kinetic rate constants *k*_*iapp*_ and *k*_*3*_ (Table 1).

3 $$\left[ {\text{P}} \right] = {\text{V}}_{{{\text{max}}}} \left( {{\text{F}}\left( {\frac{{{\text{k}}_{3} }}{{{\text{k}}_{{{\text{iapp}}}} + {\text{Fk}}_{3} }}} \right){\text{t}} + \left( {\frac{{{\text{k}}_{{{\text{iapp}}}} }}{{{\text{k}}_{{{\text{iapp}}}} + {\text{Fk}}_{3} }}} \right)\frac{1}{{{\text{k}}_{{{\text{iapp}}}} {\text{/F}} + {\text{k}}_{3} }}\left( {{\text{1}} - {\text{exp}}\left[ { - \left( {\frac{{{\text{k}}_{{{\text{iapp}}}} }}{{\text{F}}} + {\text{k}}_{3} } \right){\text{t}}} \right]} \right)} \right)$$

where F represents the ratio of free enzyme/enzyme-inhibitor complex. For potent inhibitors (*K*_*i*_ values < 100 nM, CE, PS, FS, MT, DS, PYS and CSM), the enzyme-inhibitor complex concentration is equal to the total inhibitor concentration because the *K*_*i*_ values are less than the concentration of the enzyme used in the assay (2.6 µM) by more than one order of magnitude. In this case, all inhibitor molecules are bound to an enzyme. However, for inhibitors with high *K*_*i*_ values (> 100 nM) (ES, SM, BSM, PC and TCM), not all the inhibitor molecules are bound to an enzyme, which has to be taken into account in the calculation of the *F* value. The effective concentration of enzyme-inhibitor complex was calculated using the inhibition constant formula [Eq. (4)].

4 $${K}_{i}=\left[\mathrm{E}\right]\left[\mathrm{I}\right]/[\mathrm{EI}]$$

where [E] represents the uninhibited enzyme concentration, [I] the unbound inhibitor concentration, [EI] the enzyme-inhibitor complex concentration.

#### Stoichiometry of enzyme inhibition

AHAS activity was monitored using the continuous method. For this study, CE was chosen as the inhibitor as it binds tightly to the enzyme and without accumulative inhibition in the presence of 2-mercaptpethanol. Increasing concentrations (2–6 µM) of *Afu*AHAS were incubated in the standard assay buffer with the addition 14.2 µM 2-mercaptoethanol for 15 min until maximum activity was reached. 0.5 µM of CE was then added and the enzyme activity for the first 200 s of inhibition was recorded to determine the initial inhibition rates without accumulative inhibition. A control without inhibitor was included and the measurements were performed in triplicates.

#### Fitting of the data

Fitting of data was performed using GraphPad Prism 7.01 (GraphPad Software, San Diego California USA, www.graphpad.com).

### Antifungal susceptibility assays

The antifungal susceptibility assays were conducted according to Clinical and Laboratory Standards Institute guidelines for broth microdilution M32-A2^30^, except the testing media contained Yeast Nitrogen Base, without amino acids or ammonium sulfate, buffered with 50 mM 2-[4-(2-hydroxyethyl)piperazin-1-yl]ethanesulfonic acid (HEPES). 2% glucose and 10 mM ammonium sulfate were added to the solution as the carbon and nitrogen sources, respectively. The medium was adjusted to pH 7.0 using 4 M sodium hydroxide and then stored at room temperature. 10 mg/mL stocks of each herbicide were prepared in DMSO. 10 serial twofold dilutions were conducted, giving a dilution range of 0.097656–100 µg/mL for each antifungal agent. *A. fumigatus* strain ATCC MYAA 3626 was used in this assay. The 96-well plates were incubated at 35 °C for 48 h, and the OD_530_ of each well was recorded using the Molecular Devices Spectramax 250 microplate reader (Marshall Scientific). Each experiment was performed in triplicate.

### Multiple sequence alignment

The protein sequences for the AHAS catalytic subunits were obtained from NCBI, under the accession numbers NP_013826.1 (*Sc*AHAS), NP_190425.1 (*Arabidopsis thaliana* AHAS (*At*AHAS)), EEQ44292.1 (*Ca*AHAS), AAK83371.1 (*C. neoformans* AHAS) and XP_754588.1 (*Afu*AHAS). Multiple sequence alignment was performed using T-Coffee^31^.

### Molecular modelling

The homology model of *Afu*AHAS was generated using SWISS-MODEL^21^. As the herbicide binding site conformation of *Ca*AHAS is similar to that of *Afu*AHAS, binding in *Afu*AHAS was determined by superimposing the *Ca*AHAS structures in complex with the herbicides chlorimuron ethyl (CE, PDB ID 6DEL), sulfometuron methyl (SM, PDB ID 6DEP), bensulfuron methyl (BSM, PDB ID 6DEM), penoxsulam (PS, PDB ID 6DEQ), metosulam (MT, PDB ID 6DER) and propoxycarbazone (PC, PDB ID 6DES) using Coot^32^. Energy minimization of these docked/superimposed structures was performed using the YASARA energy minimization server^33^.

## Supplementary Information

Supplementary Information.
