# Supplementary material for: Triazolopyrimidine herbicides are potent inhibitors of Aspergillus fumigatus acetohydroxyacid synthase and potential antifungal drug leads
Source: Sci Rep. 2021 Oct 26;11:21055. doi: 10.1038/s41598-021-00349-9 (PMC8548585; doi:10.1038/s41598-021-00349-9)
Supplement: Supplementary file 1 — Supplementary Information. [file 41598_2021_349_MOESM1_ESM.docx]

**Supplementary Information**


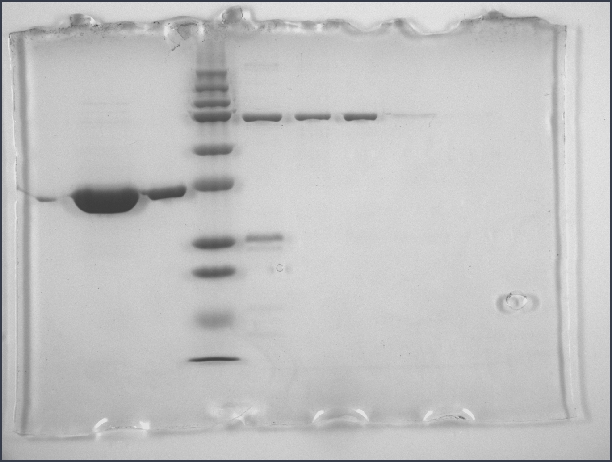


**Supplementary Figure 1.** Uncropped gel image from **Figure 2c**. The bands to the left of the molecular weight marker are for an unrelated protein.

a

b

c

d/e

f

**Supplementary Figure 2.** Effect of N_2_ bubbling and 2-mercaptoethanol on the inhibition of *Afu*AHAS by PS. The inclusion of 2-mercaptoethanol (**b**) and both 2-mercaptoethanol and oxygen removal (**c**) reduces the accumulative inhibition compared to when no treatment is included (**a**). In the control experiments, where no inhibitor was added (**d-f**) all rates were linear, but the enzyme was most active in the presence of 2-mercaptoethanol and when oxygen was depleted (**f**).**a.** Accumulative inhibition of 2.6 µM *Afu*AHAS by 10 nM PS in standard assay buffer. **b.** Inhibition of *Afu*AHAS by 10 nM PS in the presence of 14 µM 2-mercaptoethanol. **c.** Inhibition of *Afu*AHAS by 10 nM PS in the presence of 2-mercaptoethanol and N_2_ bubbling. **d.** *Afu*AHAS activity in the presence of 2-mercaptoethanol and with no inhibitor added. **e.** *Afu*AHAS activity in the absence of 2-mercaptoethanol, N_2_ bubbling and inhibitor. **f.** *Afu*AHAS activity in the presence of 2-mercaptoethanol and N_2_ bubbling with no inhibitor added. The image was created using GraphPad Prism 7.01.
